# Polycomb and Trithorax Group Proteins: The Long Road from Mutations in Drosophila to Use in Medicine

**DOI:** 10.32607/actanaturae.11090

**Published:** 2020

**Authors:** D. A. Chetverina, D. V. Lomaev, M. M. Erokhin

**Affiliations:** Institute of Gene Biology, Russian Academy of Sciences, Moscow, 119334 Russia

**Keywords:** Polycomb, Trithorax, PRE, Drosophila, PRC2, cancer, oncology, PRC2 inhibitors, EZH2 inhibitors, small-molecule inhibitors

## Abstract

Polycomb group (PcG) and Trithorax group (TrxG) proteins are evolutionarily conserved factors responsible for the repression and activation of the transcription of multiple genes in *Drosophila *and mammals. Disruption of the PcG/TrxG expression is associated with many pathological conditions, including cancer, which makes them suitable targets for diagnosis and therapy in medicine. In this review, we focus on the major PcG and TrxG complexes, the mechanisms of PcG/TrxG action, and their recruitment to chromatin. We discuss the alterations associated with the dysfunction of a number of factors of these groups in oncology and the current strategies used to develop drugs based on small-molecule inhibitors.

## INTRODUCTION

Establishment and maintenance of precise gene expression patterns that are unique to each cell type is required for the proper functioning of multicellular organisms. Transcriptional control of gene expression is one of the key steps in this type of regulation. Polycomb group (PcG) and Trithorax group (TrxG) proteins are repressors and activators of transcription, respectively [1-8]. These proteins were first characterized in *Drosophila *as regulators of *Hox *genes expression. *Hox *genes are responsible for proper body segmentation. Their baseline expression profile is determined by the protein products of the *maternal*, *gap*, *pair-rule*, and *segment polarity *genes at the early embryonic stage of development. These proteins activate each other in a cascade-like manner [9, 10, 11]. PcG/TrxG proteins were shown to be required for the subsequent maintenance of the established expression profile [12, 13].

A *Polycomb *mutation was described in *Drosophila *in 1947 [14]. Upon this mutation, the anatomical structures called sex combs, which normally form only on the first pair of male legs, also occur on the second and third pairs of legs [14]. Dysfunction of the *Polycomb *gene was shown to cause the transformation of a number of segments [15] as a result of overexpression of *Hox *genes [12, 16, 17]. In particular, sex combs result from partial transformation of the second and third pairs of legs into the first ones due to derepression of *Scr *in the *Antennapedia *complex [7]. Later, a mutation in the *trithorax *gene was discovered; its phenotypic manifestations (reduced number of sex combs) were opposite to the phenotype of *Polycomb *mutation, which is indicative of an inactivation of *Hox *genes [18, 19]. Afterwards, all mutations in other genes manifesting themselves in a manner similar to either *Polycomb *or *trithorax *were classified into the PcG and TrxG groups, respectively [4, 7]. These groups also include genes whose mutations enhance the mutant phenotypes of other known representatives as shown by genetic tests when crossing mutant flies or whose effect was demonstrated by missexpression of the *Hox *genes determined by a direct analysis.

Evolutionarily conserved PcG and TrxG proteins are found in all multicellular organisms. In mammals, mutations in the genes encoding PcG/TrxG also have a huge impact on the development of the organism [20, 21]. In addition, it was found that the area of responsibility of the PcG/TrxG proteins is much broader than only the regulation of *Hox *genes and extends to hundreds of other targets in both *Drosophila *and mammals. In particular, PcG/TrxG factors are involved in such crucial biological processes as carcinogenesis, inactivation of the X chromosome in mammals, and maintenance of the pluripotent state of stem cells [22, 23, 24].

In this review, we discuss the structure and functions of the PcG/TrxG complexes, the mechanisms of their action, and the role of individual factors in the onset, diagnosis, and therapy of oncological diseases.

## PcG AND TrxG COMPLEXES

**PcG complexes**

**Fig. 1:**
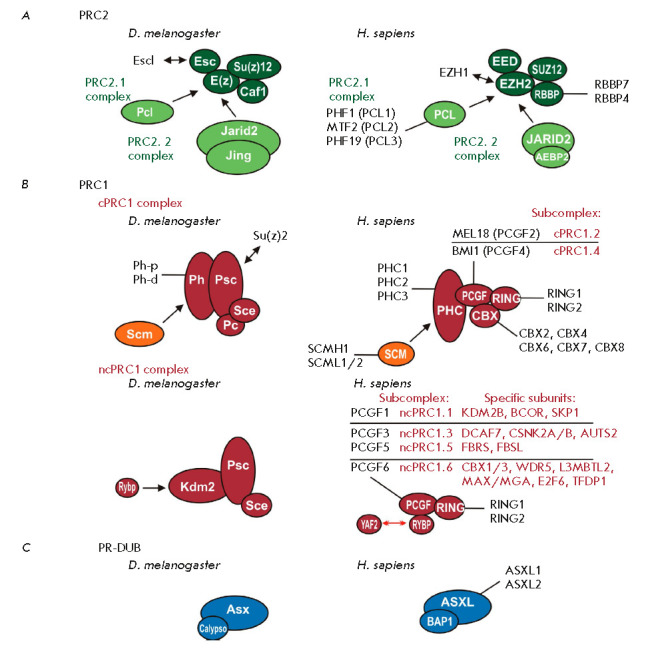
Main Polycomb group complexes. (*A*) PRC2 complexes. *Drosophila *subunits are shown on the left, and their mammalian orthologs are presented on the right-hand side of the figure. In *Drosophila *and mammals, the PRC2 core is composed of E(z)–Su(z)12–Esc–Caf1 and EZH2–SUZ12–EED–RBBP7/4, respectively. *Drosophila *Esc and mammalian EZH2 can be replaced by their homologs Escl and EZH1, respectively. The PRC2.1 complex contains either Pcl or PCL1/2/3 in *Drosophila *and humans, respectively; The PRC2.2 complex contains either Jarid2/Jing or JARID2/ AEBP2 in *Drosophila *and humans, respectively. (*B*) PRC1 complexes. The PRC1 core is composed of the Sce–Psc and RING–PCGF heterodimers in *Drosophila *and mammals, respectively. In cPRC1, the core subunits associate with Pc–Ph in *Drosophila *and their orthologs CBX–PHC in humans. In ncPRC1, the core subunits associate with Kdm2 in *Drosophila *and with the RYBP (or YAF2) subunit in humans. In humans, the cPRC1 and ncPRC1 complexes can be further distinguished by the presence of a specific PCGF subunit (cPRC1.2, cPRC1.4, ncPRC1.1, ncPRC1.3, ncPRC1.5, and ncPRC1.6 subcomplexes); other specific subcomplex subunits are indicated next to the complex name. (*C*) The PR–DUB complex. PR–DUB is composed of Asx–Calypso and ASXL1/2–BAP1 in *Drosophila *and humans, respectively. The size of the ovals representing the proteins corresponds to the relative size of the protein molecules

Most PcG proteins associate in several types of multisubunit complexes. The main complexes in *Drosophila *and mammals are PRC1 (Polycomb repressive complex 1), PRC2 (Polycomb repressive complex 2), and PR-DUB (Polycomb repressive deubiquitinase), as well as PhoRC in *Drosophila *(*Fig. 1*).

In *Drosophila*, PRC2 complexes contain the core components E(z), Esc, Su(z)12, and Caf1 [25, 26]. The Esc subunit has a homolog, Escl, which can replace it in the complex [27]. All *Drosophila *PRC2 subunits have direct homologs in mammals. However, there is only one copy of the Esc protein – the EED, and two copies of the E(z) and Caf1 factors – the EZH2/EZH1 and RBBP7/RBBP4, respectively. Protein Su(z)12 contains only one orthologue of the protein – SUZ12 [28, 29]. All the core PRC2 subunits were confirmed as PcG proteins in *Drosophila *by genetic tests [3, 4].

PRC2 mono-, di-, and trimethylates lysine 27 of histone H3 (H3K27me1/2/3) via the catalytic SET domain of the E(z) protein (EZH2/EZH1) [25, 26, 28, 29]. The H3K27me3 modification is a specific mark of the chromatin regions repressed by the PcG system [30, 31]. The lack of the H3K27me3 modification due to a point substitution of lysine to arginine at position 27 in histone H3 leads to the derepression of *Hox *genes in *Drosophila *[32].

Mammalian EZH2 has a higher methyltransferase activity than its homolog EZH1 in the *in vitro *system [33]. In addition, EZH1 plays a less important role in development: mouse embryos mutant for *EZH2, EED, *and *SUZ12 *are non-viable and die during the post-implantation period [34, 35, 36], while *EZH1 *mutants are viable and fertile [37]. In this regard, *EZH2 *and *EZH1 *have different expression profiles: higher *EZH2 *transcription is characteristic of proliferating cells, while *EZH1 *is expressed in approximately the same manner at different stages of development. However, EZH1 can replace EZH2 at later stages of development or in case of defective EZH2 [33, 38, 39].

The subunits Su(z)12/SUZ12 and Esc/EED are required for the catalytic activity of E(z)/EZH2 [26, 36, 40, 41, 42]. The interaction between Esc/EED and H3K27me3 changes the conformation of the entire PRC2 complex and stimulates its methyltransferase activity [43]. In contrast, the Caf1 subunit is not required for the methyltransferase activity of E(z) [40, 41, 42].

In *Drosophila *and mammals, the core PRC2 module can interact with additional subunits. Currently, two complexes can be distinguished: PRC2.1 and PRC2.2. The PRC2.1 complex includes the Pcl (Polycomb-like) protein in *Drosophila *and the homologous proteins PHF1, PHF19, and MTF2 in mammals. Pcl was shown to stimulate the methyltransferase activity of E(z)/EZH2 [44, 45]. The PRC2.2 complex contains the subunits JARID2 and Jing/AEPB2 [46]. JARID2 specifically binds to nucleosomes monoubiquitinated at H2AK118ub (H2AK119ub in mammals). The proteins Pcl, Jing (but not JARID2) were confirmed as PcG factors in *Drosophila *by genetic tests [3, 4].

PRC1 complexes are divided into two types: cPRC1 (canonical) and ncPRC1 (non-canonical).

*Drosophila *cPRC1 contains the core subunits Pc (Polycomb), Ph, Sce (also known as dRing), and Psc [47, 48, 49]. A Psc homolog, the Su(z)2 protein [50, 51], is co-purified with cPRC1 in non-stoichiometric amounts; it can replace Psc in the complex [52]. The *Drosophila *ncPRC1 complex, dRAF (dRing Associated Factors), contains the proteins Sce/dRing, Psc, and Kdm2 [53]. All cPRC1 and ncPRC1 subunits were confirmed as PcG proteins in *Drosophila *[3, 4].

Similar complexes are present in mammals, with Polycomb factors being represented by multiple paralogs [54, 55]. Mammalian cPRC1 includes homologous of all *Drosophila *cPRC1 subunits: Pc (CBX 2, 4, 6, 7, 8), Ph (PHC1–3), Sce (RING1/2), Psc (paralogs PCGF2 and PCGF4 in cPRC1). Mammalian ncPRCs contains Sce (RING1/2) and Psc homologs (paralogs PCGF1, 3, 5, and 6) and also the RYBP protein, which can be replaced by the YAF2 protein. Depending on the presence of one of the Psc paralogs, mammalian cPRC1 and ncPRC1 are further divided into the subcomplexes cPRC1.1, cPRC1.2 and ncPRC1.3, ncPRC1.4, ncPRC1.5, and ncPRC1.6 (*Fig. 1*). The ncPRC1.1 subcomplex, which contains a specific subunit, KDM2B (Kdm2 protein homolog), is the closest to the *Drosophila *dRAF complex. A RYBP homolog was found in *Drosophila* [56, 57]. Co-immunoprecipitation experiments have demonstrated that *Drosophila *RYBP can be co-purified with Sce and Kdm2; however, genetic tests showed that it has a double function and acts as a Trithorax factor as well [57].

The Sce/RING protein is a catalytic subunit of cPRC1 and ncPRC2 in *Drosophila *and mammals. Sce/RING possesses E3 ubiquitin ligase activity and is responsible for the H2AK118ub modification (H2AK119ub in mammals). As mentioned above, JARID2 of the PRC2.2 complex interacts with this modification. The enzymatic activity of *Drosophila *dRING in the dRAF complex is higher than that in the cPRC1 complex [53]. The cPRC1 complex can compact chromatin and repress transcription [47, 49, 58–60]. Kdm2 (KDM2B) is a histone demethylase, which removes the H3K36me2 modification characteristic of active chromatin regions [53, 61]. In addition, the Pc (CBX) protein binds nucleosomes carrying the H3K27me3 modification, which is catalyzed by PRC2 [62–64].

The Scm/SCMH1 protein, which was confirmed as a PcG factor [3], can be co-purified with *Drosophila *and mammalian cPRC1 [48, 49, 65]. In addition, Scm interacts directly with the Ph protein [66, 67]. However, at least in *Drosophila*, Scm is considered a cPRC1- independent subunit, since it can be recruited to the chromatin independently [50, 68].

The *Drosophila *PR–DUB (Polycomb repressive deubiquitinase) complex consists of the Calypso and Asx proteins [69]. Calypso is a deubiquitinase that removes the H2AK118/9ub modification, while Asx stimulates the enzymatic activity of Calypso [69]. Despite the fact that Calypso and Asx have a function opposite to the PRC1 complexes, they act as PcG factors. Mammals have two complexes with similar activity. Both complexes have a homolog of Calypso (BAP1) and include one of the Asx protein homologues – ASXL1 (which forms the PR–DUB1 complex with BAP1) or ASXL2 (which forms the PR–DUB2 complex with BAP1) [54]. The role of the simultaneous presence of ubiquitinase and deubiquitinase specific to the same histone H2A amino acid is currently unknown.

The PhoRC complex is a DNA-binding PcG complex, which includes Sfmbt and Pho [70]. Both factors are PcG proteins; their mutants are characterized by derepression of *Hox *genes [70, 71]. Pho contains a DNA-binding domain composed of C2H2-type zinc finger motifs. A Pho homolog, the Phol protein, shares the same DNA-binding site with Pho [72] and can interact with Sfmbt, instead of Pho [70]. Unlike Pho, Phol mutants do not exhibit a homeotic phenotype. Genome-wide distribution of Pho is different from that of Phol: while the main Pho peaks overlap with the PRC1 and PRC2 proteins, Phol major peaks are at the promoters of active genes [73]. Meanwhile, both factors are involved in the recruitment of PcG proteins to the chromatin (see below).

Mammals have direct homologs of the PhoRC complex subunits. However, attempts to isolate this complex have remained unsuccessful so far. The YY1 protein is a Pho/Phol homolog, while the proteins L3MBTL2, MBTD1, and SFMBT1 are Sfmbt homologs. Moreover, the YY1 protein retains the region necessary for Pho to interact with Sfmbt in *Drosophila*. *In vitro *experiments have shown that this region can interact with L3MBTL2, MBTD1, and SFMBT2. However, this interaction is 50- to 100-fold weaker than that of the Pho-Sfmbt interaction in *Drosophila *[74]. This may explain the fact that YY1 wasn’t detected upon purification of the L3MBTL2 complex by co-immunoprecipitation [65, 75, 76]. Moreover, YY1 is associated with RYBP (YY1–RYBP complex) in mammals. YY1–RYBP was shown to participate in both the repression and activation of the transcription of a large number of genes [77, 78].

**TrxG complexes**

The TrxG is a more heterogeneous group of proteins than PcG, and genetically identified TrxG factors are subunits of different complexes involved in transcription activation [3, 4, 7, 8]. Describing the complexes, we indicate the TrxG factors that were identified by genetic tests in *Drosophila*; i.e., the factors whose mutations have phenotypes opposite to *Polycomb *mutations.

**Fig. 2:**
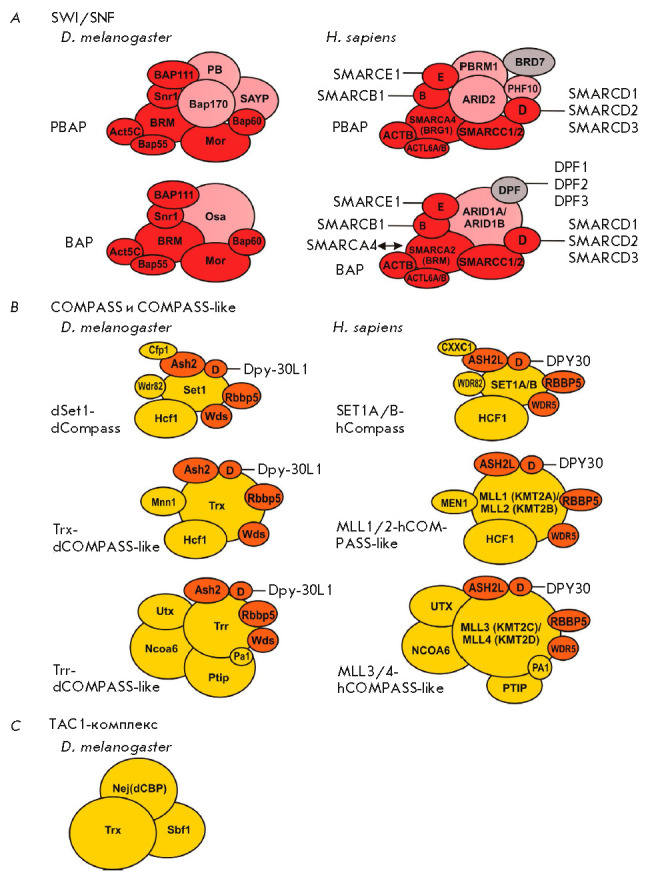
Main Trithorax group complexes. (*A*) The BAP and PBAP complexes of the SWI/SNF subfamily. The subunits common to both complexes are colored in red; specific BAP and PBAP subunits are shown in pink. Human subunits whose presence in *Drosophila *BAP/ PBAP complexes has not been confirmed are depicted in grey. (*B*) COMPASS and COMPASS-like complexes. The subunits common to the three complexes are colored in orange; the specific subunits are presented in yellow. (*C*) *Drosophila *TAC1 complex (not confirmed as present in humans). The size of the ovals representing proteins corresponds to the relative size of the protein molecules

A number of Trx group factors are subunits of the ATP-dependent chromatin remodeling complexes (*Fig. 2*). Using the energy of ATP hydrolysis remodelers alter the structure, assembly, and position of nucleosomes on the DNA and, thus, facilitate the recruitment of activator complexes to the chromatin [79]. Five proteins – Osa, Brm, Mor, Snr1, and SAYP – which behave as TrxG factors in genetic tests in *Drosophila*, were shown to be subunits of the SWI/SNF subfamily of the ATP-dependent chromatin remodeler complexes: BAP (Brahma-associated proteins) and PBAP (Polybromo-associated BAP) [80-83]. The Brm (ATPase), Mor, and Snr1 proteins are subunits common to both complexes, while the Osa and SAYP proteins are specific to BAP and PBAP, respectively. All these TrxG factors have homologs in mammals which form similar complexes [79]. The Brm protein has two homologs, named SMARCA2 and SMARCA4. PBAP contains only SMARCA4, while the BAP complex can contain both homologs. Mor is homologous to the SMARCC1 and SMARCC2 proteins; Snr1 is homologous to SMARCB1. Like in *Drosophila*, homologs of the SAYP (PHF10) and OSA (ARID1A and ARID1B) factors are specific to PBAP and BAP, respectively.

The Trithorax (Trx) protein, which gave the name to the entire group, is a histone H3K4 methyltransferase. The Trx has two homologs in *Drosophila*: the Trr (Trithorax-related) and Set1 proteins. The direct mammalian orthologs of these proteins are SET1A and SET1B (Set1), MLL1 and MLL 2 (Trx), and MLL3 and MLL4 (Trr) [21, 84, 85]. All three factors and their orthologs were shown to form similar complexes: COMPASS and COMPASS-like. All complexes share common subunits: Ash2 (ASH2L), Dpy–30L1 (DPY30), Rbbp5 (RBBP5), and Wds (WDR5). In *Drosophila*, the Ash2 protein is a TrxG factor confirmed by genetic testing. These complexes catalyze H3K4me1/2/3-specific methylation of nucleosomes, which is characteristic of active chromatin regions [8, 21, 86]. According to a number of publications, the H3K4me2 modification is associated with both enhancers and gene promoters; H3K4me3 is associated with promoters of actively transcribed genes, while H3K4me1 has higher specificity to enhancers [87]. In addition, the modifications H3K4me1 and H3K4me2 in *Drosophila *overlap with the known sites of PcG complexes recruitment to chromatin (PRE, see below) [88, 89]. According to current data, Set1 is responsible for the majority of the di- and trimethylation of H3K4 in *Drosophila *cells [90, 91], while the main function of the Trx/Trr proteins is monomethylation of H3K4 [89, 92]. Similarly, the mammalian proteins MLL1/MLL2/MLL3/MLL4 mediate H3K4me1 [89, 93]. It is noteworthy that UTX, which is a subunit of Trr (MLL3/MML4)-Compass-like, is a H3K27me2/3 demethylase [94-99].

The TAC1 complex contains the TrxG factor – histone acetylase dCBP (also known as Nejire) – and the Sbf1 protein [100]. Mammals have two homologs of the Nej protein: P300 (EP300) and CBP (CREBBP). However, a TAC1-like complex has not yet been characterized. The Proteins dCBP/P300/CREBBP catalyze the H3K27Ac modification in active chromatin [101, 102]. Acetylation of histones weakens the interaction between nucleosomes and DNA and leads to chromatin decompaction [103]. The dCBP, Trx, and Trr proteins, as well as the modifications H3K4me1 and H3K27Ac catalyzed by them, respectively, were shown to co-localize at active enhancers and at the regions of PcG proteins recruitment in *Drosophila*. Moreover, acetylation of H3K27 *in vitro *is enhanced in the presence of H3K4me1, Trr, and Trx [89, 92, 101].

Another protein genetically characterized as TrxG is the Ash1 protein, which, like its mammalian homolog ASH1L, methylates histone H3 lysine 36 (H3K36me2) [104, 105, 106] and, respectively, has an activity opposite to that of Kdm2 (KDM2B). Furthermore, effective methylation of H3K36 requires the TrxG protein Kis [107], which is a homolog of the ATP-dependent chromatin remodeling proteins belonging to the CHD subfamily in mammals [108].

Rad21 (RAD21 in mammals) [109], a subunit of the cohesin complex, also belongs to the Trithorax group of proteins identified by genetic tests. The cohesin complex stabilizes long-range interactions in the nucleus, including enhancer-promoter contacts, which are necessary for the activation of transcription [110, 111].

## MECHANISMS OF POLYCOMB AND TRITHORAX COMPLEXES RECRUITMENT TO CHROMATIN

**PRE elements in Drosophila**

Specific DNA elements that serve as PcG binding fragments were found in the *Drosophila *genome: Polycomb Response Elements (PRE) [3, 112-115]. PRE elements can be located both at a distance from the target gene (tens and hundreds of thousands of base pairs) and in the immediate vicinity of the transcription start site (TSS).

PREs were shown to act as the memory elements of the repressed state; they ensure a proper enhancer activity profile established at the early stages of embryogenesis. In transgene constructs outside of the genomic environment (in the absence of PRE elements), embryonic enhancers exhibit proper segment-specific activity only at the early stages of development (0–6 hrs), after which they become active in other parasegments, where they are normally inactive. However, a nearby inserted PRE element can maintain the correct pattern of enhancer activity at later stages of embryogenesis and suppress gene activation in unnecessary segments [116] (see details in reviews [3, 115]).

It was shown that PREs lack predetermined tissue specificity and that the enhancer determines the region of PRE activity. A number of studies have shown that PRE can either be switched from the repressing to activating state or become inactivated in the presence of an activator/enhancer. The dual nature of PREs can be also witnessed in a series of transgenic lines that carry the same transgenic construct inserted in different regions of the genome. It turns out that PRE activity is very sensitive to the insertion site, and that repression is observed only in half of the lines. In non-repressing cases, PREs presumably either exist in a neutral state or activate transcription. In addition, a number of embryonic enhancers that regulate developmental genes possess the PRE property in adults [117], indicating that at least some PREs in the activating state can potentially act as classical enhancers. In accordance with their dual activity, PREs can recruit not only PcG but TrxG proteins as well. It is important to note that PcG/TrxG proteins can be recruited to PRE DNA regardless of the PRE state [30, 118, 119, 120], which suggests direct competition between the PcG/ TrxG proteins in PREs functioning. In accordance, the core subunits of the PcG complexes can be associated with active chromatin regions and potential enhancers [121-125]. It is believed that PcG proteins can inhibit the excessive activity of enhancers and promoters in these regions.

**Recruitment of PcG/TrxG proteins to PRE elements**

The minimum length of the PRE element required for the repression of reporter genes in transgenes is several hundred base pairs. For instance, the minimum DNA fragments sufficient for repression are 217 bp, 181 bp and 152 bp in case of *Fab7*PRE, *en*PRE and *eve*PRE, respectively [126, 127, 128].

The core PRE sequences contain sites for various DNA-binding factors. The characterized PRE DNA-binding factors in *Drosophila *are the Pho, Phol, GAF, Combgap, Spps, Zeste, Psq, Adf1, Grh, and Dsp1 proteins [112, 113]. The exact combination, number, and relative position of these protein binding sites vary in different PREs, which indicates their unequal role in the functioning of an individual PRE. It was shown that 90% of PRC1/PRC2 binding peaks overlap with Pho and Combgap [129-132]. Half (50%) of the GAF and Dsp1 peaks overlap with PRC1 [132]. The Zeste and Phol colocalize with approximately 25% and 21% of PRC1 peaks, respectively [132].

It is important to note that most, if not all, DNA-binding factors associated with PRE participate in both repression and activation of transcription and have other targets in the genome, including promoters of active genes and potential enhancers [112, 113]. Except for Pho, mutations in the genes encoding the proteins of this group do not have a clear PcG phenotype. It is important to note that the binding sites responsible for the recruitment of a single DNA-binding protein, including Pho, as well as the combinations of the sites for different proteins, cannot ensure the recruitment of PcG proteins. This suggests the existence of a combinatorial component in the functioning of PREs, in which DNA-binding proteins form a platform for the recruitment of PcG proteins [112].

Despite the fact that the analyzed combinations of DNA-binding sites do not recruit PcG factors, these proteins/or their binding sites are significant in functional tests [112]. The role of Pho and its homolog Phol in the recruitment of PcG proteins is the best studied. Inhibition of Pho by RNA interference (RNAi) in a *Drosophila *cell line lacking Phol expression was shown to diminish the binding of Pc (PRC1), E(z), and Su(z)12 (PRC2) to one of the well-characterized PRE (*bxd*PRE) [68, 133]. At the larval stage, when both homologs are expressed, inactivation of both factors is required for the loss of PcG proteins binding [133]. The factors Pho/ Phol were found to establish direct contacts with PRC1 and PRC2. Pho interacts directly with the E(z), Esc (PRC2) [133] and Ph, Pc (PRC1) proteins [134], while Phol associates with Esc [133]. The dependence of PcG protein recruitment on Pho/Phol varies between different PREs. For example, a genome-wide study has demonstrated that, in addition to Pho, the DNA-binding factors Spps and Combgap play an important role in the recruitment of PcG proteins to a number of PREs [125, 135].

PRE functioning, the Spps, Dsp1, GAF, and Grh proteins can foster interactions between Pho and PcG [125, 135-138].

Grh was shown to interact directly with Sce (PRC1) [139] and Pho [136]. According to two-hybrid screening results, Spps directly interacts with Scm [140], which, in turn, can associate with the proteins Ph [66, 67] and Sfmbt [141, 142]. These interactions can stabilize the recruitment of PcG proteins to the chromatin.

In addition to the recruitment of PcG proteins, DNA-binding factors can participate in the binding of TrxG proteins to PRE. Pho was shown to interact directly with Brm ATPase [134], Zeste associates with MOR [143], while GAF is required for the recruitment of Brm and Polybromo to *bxd*PRE [144].

Thus, DNA-binding proteins in *Drosophila *can recruit both Pc and Trx group proteins. Apparently, the commonality of DNA-binding factors between the PcG/TrxG complexes increases the plasticity of transcription regulation processes by facilitating, if necessary, a rapid switch from repression to activation and vice versa.

**PRE elements in mammals**

A number of PRE-like elements, as well as a number of DNA-binding proteins associated with the PcG/TrxG complexes, have been described in mammals [145-149]. Among DNA-binding proteins are the AEBP2, REST, SNAIL, RUNX1, E2F6, and MGA/MAX factors [1].

However, it should be noted that in mammals, in addition to sequence-specific DNA-binding proteins, a large role in PRC2 recruitment belongs to the CpG islands (CGI) [150, 151, 152].

Apparently, as in the case of the *Drosophila *genome, there is no universal DNA-binding recruiter responsible for the binding of all of the Polycomb or Trithorax proteins to the chromatin. The existence of numerous PcG paralogs indicates the possibility of a wide variety of DNA-binding factors as well. This, taking into account the tendency of DNA-binding factors to partially functionally substitute for each other, creates obstacles for their identification. We suppose that combinations of binding sites for different DNA-binding proteins can play the primary role in the recruitment of the PcG/TrxG factors to the chromatin that form fairly extended regions for stable PcG/TrxG recruitment.

**Epigenetic modifications**

A number of studies indicate the impact of nucleosome modifications on the recruitment of Polycomb/Trithorax complexes. Methyltransferase E(z)/EZH2/EZH1 of the PRC2 complex creates the H3K27me3 modification, which is bound by the Pc/CBX protein of the PRC1 complex. On the other hand, the Sce/RING subunit of the PRC1 complex mediates H2AK118/9ub that is recognized by the JARID2 subunit of the PRC2 complex. The identified activities and interactions suggest the existence of positive feedback facilitating the recruitment of the PRC1 and PRC2 complexes to the chromatin. However, disruption of the PRC2 activity does not completely eliminate the binding of PRC1 subunits [153, 154]. This indicates that histone modifications can rather increase the affinity of PcG complexes to the chromatin than serve as the main recruitment factor. The role of the H2AK118/9ub modification is of great interest. Impaired ubiquitination activity of Sce/RING1 in *Drosophila *and mice does not lead to a significant loss of Polycomb-dependent repression [155, 156]. However, it should be indicated that interrelation between PRC1 and PRC2 recruitment can depend on the object of study, since it has recently been shown that elimination of the catalytic activity of RING1 leads to a significant loss of PRC2 binding in mouse embryonic stem cells [157, 158]. Moreover, binding of the JARID2-containing PRC2.2 complex, which specifically associates with the H2AK119ub modification, was affected more strongly compared to the PRC2.1 variant, which contains PCL.

**Long Non-coding RNAs**

Long, non-coding RNAs (lncRNAs) are found at many mammalian genomic loci regulated by Polycomb repressors. Mutations in the Polycomb group genes were shown to suppress the activity of some lncRNAs. For instance, damage to the PRC2 core component, the EED protein, disrupts the activity of Xist lncRNA, which is required for X-chromosome inactivation in mammals [159], and of the lncRNAs involved in genomic imprinting [160]. This has led to the hypothesis that the fundamental step in the recruitment of Polycomb group repressors is the binding of PRC2 to non-coding RNAs that can attract this complex to chromatin [161]. However, it was subsequently established that PRC2 can associate randomly with various RNAs, including short RNAs, actively transcribed mRNAs, and even bacterial RNAs [162, 163, 164].

Recent studies have shown that the non-canonical PRC1 complex containing PCGF3/5 components can interact with Xist lncRNA [165, 166, 167]. This interaction is mediated by the RNA-binding factor hnRNP K, which efficiently recognizes C-rich motifs in RNA [168]. These data suggest a more specific binding of PRC1-Xist in comparison with PRC2-Xist (discussed in [169]). However, it has not been established whether this mechanism can be extended to the recruitment of Polycomb factors in the case of other regions of the mammalian genome and lncRNAs.

Attempts have also been made to elucidate the potential role of lncRNAs in *Drosophila*. However, no stable association of lncRNAs with PRC1 and PRC2 has yet been found.

Summing up, it can be assumed that in *Drosophila, *as well as in mammals, the DNA-binding factors and specific combinations of their binding sites play an important role in the targeted recruitment of Polycomb/ Trithorax proteins to chromatin. Currently, especially in the mammalian genome, there is only limited information about PcG-associated DNA-binding factors and identification of these factors is one of the important tasks in the near future [170]. Epigenetic modifications of histones (and DNA modifications in mammals), as well as RNA-protein interactions, can play an important role in stabilizing interactions between Polycomb/ Trithorax factors and chromatin. However, the specificity of a set of genomic targets is, apparently, determined by particular DNA sequences and the proteins that bind them.

## MECHANISMS OF POLYCOMB/TRITHORAX PROTEINS ACTION

**Competition between the PcG and TrxG proteins**

**Fig. 3:**
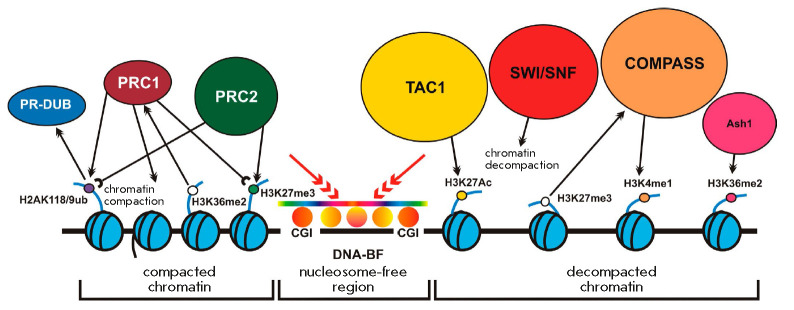
Functional activities of the Polycomb/Trithorax group proteins. The PRC1 complex compacts chromatin, mediates nucleosome ubiquitination (H2AK118Ub in *Drosophila*, H2AK119Ub in mammals), and also specifically binds to the nucleosome tri-methylated at H3K27. The PRC2 complex is responsible for the H3K27me3 histone modification; it interacts with the H2AK118/9Ub nucleosomes. The PR-DUB complex deubiquitinates H2AK118/9Ub nucleosomes. Trithorax group activator complexes decompact chromatin (SWI/SNF), acetylate histones (TAC1), and catalyze H3K4 methylation (COMPASS). DNA-binding factors (DNA-BF) and hypomethylated CpG islands (CGIs) are involved in the recruitment of the PcG/TrxG complexes to chromatin

Many known functions of the TrxG proteins counteract the activities of the PcG proteins (*Fig. 3*). Competition between PcG and TrxG proteins can occur at the PREs, enhancers, and gene promoters.

TrxG activators mediate the H3K36me2 modification [104, 105, 106], H3K27me3 demethylation [94-99], and the acetylation of H3K27 [89, 101, 102].

PcG repressors catalyze the H3K27me3 modification [25, 26, 28, 29] and demethylation of H3K36me2 [53, 61]. In addition, a number of studies indicate that PcG proteins can function in tandem with the histone deacetylase Rpd3/HDAC1 which is responsible for the deacetylation of H3K27Ac [171, 172, 173].

Histone modifications that are markers of active chromatin were shown to inhibit PcG modifications. For instance, the modifications H3K4me3 [174], H3K36me2/3 [106, 174], and H3K27Ac [101] inhibit the methylation of H3K27me3.

The competition between PcG and TrxG proteins also influences the chromatin structure. While PRC1 can compact chromatin [58], the TrxG BAP and PBAP complexes, as well as the acetylation of nucleosomes by CBP, promote chromatin decompaction [79, 103].

**Spatial interactions and PcG/TrxG function**

In multicellular organisms, nuclear DNA is organized into highly ordered structures that possess several levels. The first level of DNA packaging is the nucleosomes, which are assembled into chromatin fibers. At a higher level, the fibers form loop structures folded into topologically associated domains (TADs). Interactions between TADs lead to the formation of active and inactive chromosome compartments, which are partitioned into chromosome territories [175-178]. Individual genomic loci located at a great distance from each other on the same chromosome or even different chromosomes can physically interact with each other.

Some of the first evidence of the importance of spatial interactions in the activity of PRE/TRE elements was obtained on transgenic *Drosophila *lines. In the lines, repression of the marker gene by PRE increased in flies homozygous for the transgenic construct. This effect, known as Pairing Sensitive Silencing (PSS), is assumed to depend on the ability of the two PREs copies located on homologous chromosomes to interact and enhance each other’s activity [179, 180]). In addition, PRE elements are able to repress target genes at a long distance, and this activity can be blocked by insulators [181, 182, 183]. In this aspect, the PRE/TRE is akin to the activity of enhancers, which are also able to initiate long-distance and ultralong-distance interactions with the target gene promoters, all this regulated by insulator elements [3, 184].

## DYSFUNCTION OF POLYCOMB/TRITHORAX PROTEINS IN ONCOLOGICAL DISEASES

Misregulation of the activity of PcG/TrxG factors has been described in many pathological conditions, including cancer. The proteins of these groups play an important role in various cellular processes and can act as either tumor suppressors or oncogenes, depending on the tumor and tissue context. It has been shown that violation of at least one of the BAP/PBAP subunits occurs in about 25% of cancers [185]. An essential role in carcinogenesis was also revealed for H3K4-specific methyltransferases of the COMPASS and COMPASS-like complexes [186, 187]. The role of PRC1 complex PcG factors in carcinogenesis and the possibility of creating small-molecule inhibitors to block their activity are under active studies [22, 188].

In this review, we focus on the role of the PRC2 complex in oncological diseases. Studies in the last decade have demonstrated a wide variety of changes in EZH2 and its partners in cancer. This has led to the development of a number of small-molecule inhibitors to block PRC2 activity. One of them, tazemetostat, was approved in January 2020 for clinical use in medical practice in the United States [189, 190, 191].

**Dysfunction of the activity of the PRC2 complex in carcinogenesis**

It is now a demonstrated fact that both enhancement and suppression of PRC2 activity can lead to cancer. Basically, changes are detected in the core PRC2 subunits: the EZH2, SUZ12, and EED proteins [21, 23, 192, 193, 194]. The most studied to date case is the enhancement of PRC2 activity, i.e., situations in which PRC2-encoding genes act as oncogenes. In many types of malignant tumors, overexpression of PRC2 components is observed. Activation of the PRC2 function can also be a result of gain-of-function (GOF) mutations, which increase the catalytic activity of EZH2/EZH1. On the other hand, tumors associated with *EZH2-*, *SUZ12-*, or *EED*-impaired activity have also been described, which suggests a tumor-suppressive role for PRC2 in these cases.

**Oncogenic role of PRC2**

**Fig. 4:**
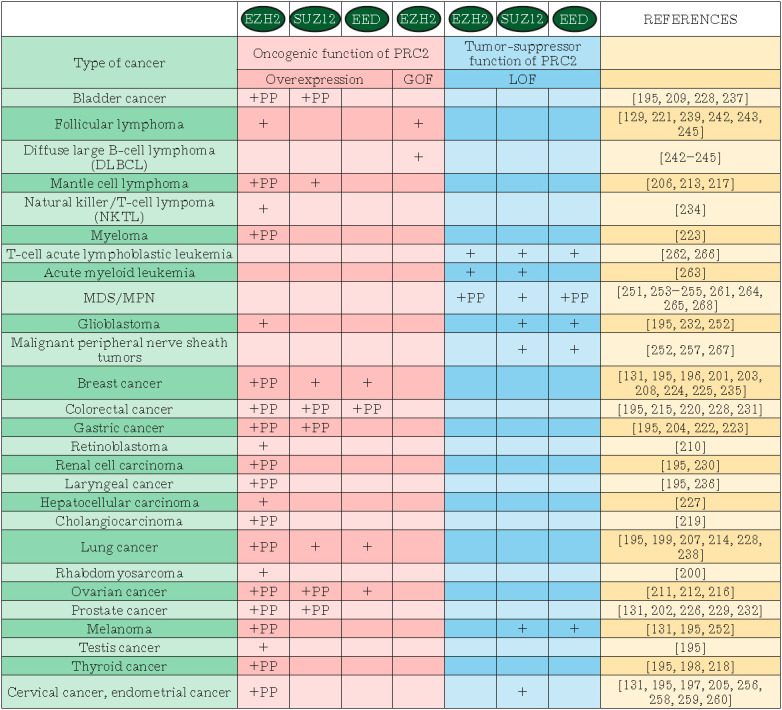
Disruption of the PRC2 core subunits’ activity in carcinogenesis. The “+” sign against the pink background stands for cases of hyperactivation of the PRC2 enzyme (overexpression or GOF mutations); “+”against the blue background stands for cancer associated with a loss of the PRC2 function (LOF mutations); “PP” (Poor Prognosis) indicates that the PRC2 subunit dysfunction was shown to correlate with a poor survival prognosis. MDS/MPN – myelodysplastic/myeloproliferative neoplasm

*Overexpression of EZH2, SUZ12, and EED. EZH2 *is the gene whose transcription level changes most commonly during carcinogenesis, compared to other PRC2 subunits. In normal cells the transcription level of *EZH2 *is regulated by the RB–E2F signaling pathway, and the high level of *EZH2 *expression in proliferative cells is significantly lower in differentiated cells [33, 195, 196]. However, *EZH2 *overexpression is observed in many malignant neoplasms [131, 195, 197-236] (*Fig. 4*). *EZH2 *overexpression was found to be associated with an increased level of H3K27me3 and, often, associated with an amplification of the EZH2-encoding gene [195, 221, 226]. In some cases, a correlation between a high expression of *EZH2 *and poor survival prognosis was noted [131, 197, 199, 202, 204, 206-209, 212, 213, 214, 218, 219, 222, 223, 224, 225, 227-231, 236].

Overexpression of *SUZ12 *and *EED *was detected in some types of cancer as well [202, 209, 211, 212, 214, 215, 217, 233, 237, 238]. However, there is currently significantly less clinical data regarding these genes. In a number of studies, an increased level of *SUZ12 *and *EED *transcription is associated with poor survival prognosis [202, 209, 212, 215, 233, 237].

*EZH2 GOF mutations. *In addition to *EZH2*, *SUZ12*, and *EED *overexpression, the activity of the PRC2 complex can be enhanced by GOF mutations in the methyltransferase domain of *EZH2*. Such mutations have been described in specific types of non-Hodgkin lymphomas (diffuse large B-cell lymphoma (DLBCL) and follicular lymphomas) [129, 221, 239-245] (*Fig. 4*). Point Y641→F,N,H,S substitutions relative to isoform C (denoted as Y646 relative to isoform A) are the most common mutations [129, 221, 239, 242-245]. There are also functionally similar mutations at the A677 and A687 positions [129, 221, 241, 243, 246, 247].

Mutant forms of EZH2 were shown to more efficiently methylate histone H3 (H3K27me2), which leads to an increased degree of H3K27me3 modification. In lymphoid tumors with monoallelic GOF mutations, wild-type EZH2 prefers H3K27me0/me1 nucleosomes as a substrate for methylation, while the mutant form shows enhanced catalytic activity against H3K27me2 [248, 249]. However, GOF mutations in *EZH2*, despite their widespread occurrence in lymphomas, are not associated with a poor survival prognosis in follicular lymphomas [239] and DLBCL [244].

GOF mutations were also detected in the EZH2 homolog EZH1 (Q571R) in thyroid adenoma [250]. This mutation also results in an increased level of H3K27me3.

**Tumor-suppressive role of PRC2**

*LOF mutations in the PRC2 complex subunits. *LOF (loss-of-function) mutations that disrupt PRC2 activity have been described in all three core components: EZH2, SUZ12, and EED [251-268] (*Fig. 4*). LOF mutations in *EZH2 *and *EED *have been shown to be associated with a negative prognosis in myelodysplastic syndrome/myeloproliferative neoplasm [251, 253, 254, 255, 261, 265, 268].

Thus, the consequences of an inactivation of the PRC2 function observed in a number of tumors remain insufficiently studied, while the data on PRC2 hyperactivity are more substantive. Further studies will help elucidate the significance and frequency of LOF PRC2 mutations in different types of tumors.

*H3K27M mutation of histones. *Another type of mutations affecting the activity of PRC2 are point substitutions in the *H3F3A *and *HIST1H3B *genes (which encode for the histone variants H3.3 and H3.1, respectively). These mutations lead to the substitution of lysine for methionine at position 27 of H3 (H3K27) and are designated as H3K27M. It was found that such mutant histone variants interact with EZH2 and inhibit the methyltransferase activity of the PRC2 complex, decreasing the H3K27me3 level both *in vivo *and *in vitro *[269-272]. H3K27M mutations are found in 80% of pediatric gliomas [273, 274, 275] and in 6% of secondary acute myeloid leukemias [276]. It was recently demonstrated that EZH2 can be automethylated at positions EZH2-K510 and EZH2-K514. This automethylation stimulates the histone activity of EZH2 and is impaired in lines carrying the H3K27M mutation [277].

Suppression of PRC2 activity by the factor EZHIP (EZH2 Inhibitory Protein) has recently been discovered in ependymoma cells (CNS tumor) [278-281]. The EZHIP region is considered to mimic the H3K27M structure and inhibit PRC2 activity in a similar way.

**Mechanisms of the oncogenic and tumor-suppressive PRC2 roles**

The mechanisms that underlie the opposite PRC2 roles in different types of tumors are currently being studied vigorously. In general, these differences are driven by the PRC2-mediated suppression of either oncogenes or tumor suppressors in different type of cells.

Overexpression of *EZH2 *has been shown to enhance cell proliferation both *in vitro *[195, 208] and *in vivo *[282, 283, 284]. An increased EZH2 level stimulates metastasis [285], cell invasion [208], and affects DNA repair [283]. GOF mutations in *EZH2 *accelerate MYC- and BCL-2-mediated lymphomagenesis in mice [282, 286]. The available data indicate that the oncogenic effect of PRC2 consists in inhibiting the transcription of a number of tumor suppressors, while the specific set of tumor suppressors to be inhibited is strongly dependent on the type of tumor. For instance, PRC2 suppresses *CDKN2A *transcription in prostate and endometrial cancer cells, as well as in lymphoid tumors (see details in [192]). It should be noted that PRC2 inactivation suppresses the growth of some tumor cells *in vitro *and *in vivo*, which has allowed for the development of small-molecule inhibitors (see below for details).

The mechanisms of tumor-suppressive PRC2 action have been less studied. However, such PRC2 targets as, for instance, the oncogenes *HOXA9 *and *MYC *are overexpressed in many types of tumors [252, 255, 262, 287]. In transgenic mice, somatic deletions of *EZH2 *and *EED *interact with the Q61K mutation of the *NRAS *oncogene and hyperactivate the STAT3 signaling pathway, leading to acute myeloid leukemias [288]. The combination of mutations in the *EZH2*/*RUNX1 *or *EZH2*/ *p53 *gene leads to the formation of therapy-resistant myeloid lymphocytic leukemias in mouse models [289, 290]. A *SUZ12 *deletion interacts with the JAK3 factor mutation, leading to acute lymphoblastic T-cell leukemia [291]. Inactivating mutations in *EZH2 *contribute to the development of a myelodysplastic syndrome that is induced by mutations in *RUNX1 *[292]. The loss of SUZ12 activity is associated with a *NF1 *mutation in tumors of the peripheral nervous system: glioma and melanoma [252]. *NF1 *encodes a GTPase, which activates the *ras *gene; and mutations in this factor result in Ras-dependent activation of carcinogenesis [293]. A loss of PRC2 activity is also observed in the case of other gene disfunctions: for example, mutations in *ASXL1 *[294], or upon HMGN1 overexpression [295] in leukemia.

Thus, depending on the mutations or changes in the expression of other genes, PRC2 inactivation can lead to malignant cell transformation [296, 297]. Carcinogenesis can be associated with a loss of function by all three core components of the PRC2: EZH2, SUZ12, and EED.

**Small-molecule PRC2 inhibitors**

**Fig. 5:**
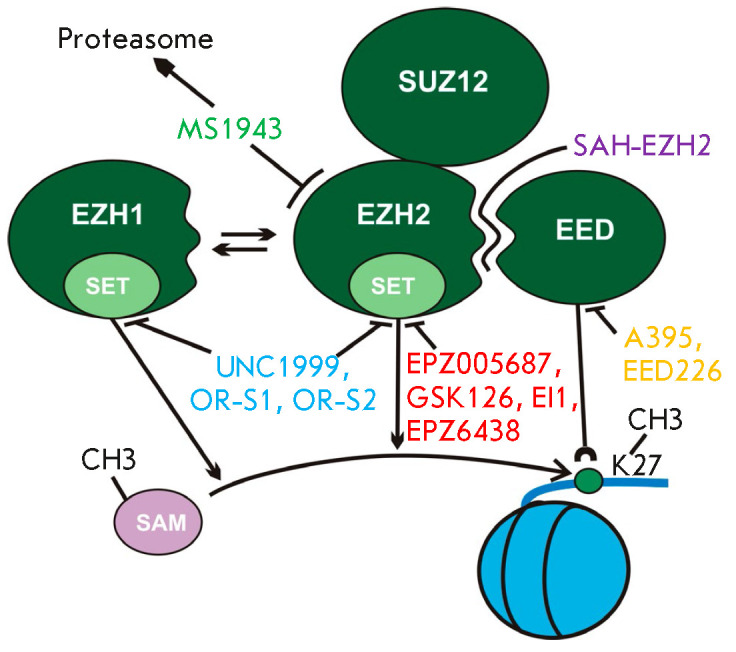
Schematic representation of the mechanisms of suppression of PRC2 hyperactivity by small-molecule inhibitors. **EPZ005687, GSK126, EI1, **and **EPZ6438 (tazemetostat) **target the EZH2 SET domain and inhibit the transfer of a methyl group from S-adenosylmethionine (SAM) to histone H3 lysine 27 (H3K27)**. UNC1999, OR-S1, **and **OR-S2 **suppress the activity of EZH2 and its close homolog, EZH1. **SAH-EZH2 **inhibits the interaction between EZH2 and EED, which leads to destabilization of the PRC2 complex. **A-395 **and **EED226 **suppress the recruitment of the EED protein to the H3K27me3 modification, eliminating the stimulation of the PRC2 methyltransferase activity. **MS1943 **recognizes the unique three-dimensional structure of the EZH2 protein and directs it to the proteasome degradation pathway

The discovery of numerous abnormalities associated with PRC2 hyperactivity stimulated scientists to develop small-molecule inhibitors that suppress the activity of this complex (*Fig. 5*).

The first of such substance was DZNep. This inhibitor reduced the level of H3K27me3 modification in tumor cell cultures [298]. However, it was later found that treatment of cells with DZNep decreases the overall level of nucleosome methylation at different positions [299]. At the next step, three inhibitors, named EPZ005687 [300], GSK126 [301] and EI1 [302], were developed. They specifically inhibited the methyltransferase activity of both the native and GOF mutant (at position Y641) forms of the EZH2 protein. The mechanism of these inhibitors’ action is based on the competition with the cofactor S-adenosylmethionine (SAM) for selective binding to the SET domain of EZH2. The treatment of a cell culture with the EI1 inhibitor has a comparable effect on the level of H3K27me3 as the complete deletion of the *EZH2 *gene [302], while the GSK126 is able to suppress the *in vivo *growth of a tumor obtained by xenotransplantation of human lymphoma KARPAS422 cells in mice [301].

The EZH2 inhibitor EPZ-6438 (which was later registered as Tazemetostat) also targets the methyltransferase domain of EZH2. The activity of EPZ-6438 has been demonstrated on malignant rhabdoid tumors (MRTs). These cells carry the mutant *SMARCB1 *gene, which encodes the subunit of the SWI/SNF chromatin remodeling complex [303]. Mutations in this gene are often found in rhabdoid tumors [304] and confer a high sensitivity of the tumor cells to the suppression of the PRC2 activity [305]. Treatment of xenograft-bearing mice with EPZ-6438 was shown to decrease the total level of H3K27me3 and reactivate a number of repressed genes. Further experiments also confirmed the ability of EPZ-6438 to suppress the proliferation of tumor cells derived from lymphoid tumors [306].

All of the described inhibitors were highly specific to EZH2 and much less active against EZH1. However, it had previously been shown that EZH1 can replace EZH2 if the latter is damaged. Thus, in some cases, when using highly specific inhibitors that block the activity of EZH2, the PRC2 complex can retain partial activity thanks to EZH1. Therefore, a series of inhibitors were further developed to solve this problem.

The inhibitors UNC1999 [307], OR-S1, and OR-S2 [308, 309] target the methyltransferase activity of both EZH2 and EZH1. Suppression of cell proliferation by OR-S2 was analyzed on a big set of tumor cell lines [309]. OR-S2 was shown to inhibit the growth of 33 out of 68 tumor cell lines of hematopoietic origin (lymphoma, myeloma, and leukemia) and 26 out of 124 solid tumors. The cytotoxic effects of OR-S1 and OR-S2 have also been confirmed on models of gastric cancer, rhabdoid tumors, and acute myeloid leukemia [308, 309].

High-throughput screening methods can be used to search for the novel small-molecule inhibitors of PRC2. For example, screening of approximately 250,000 substances allowed scientists to identify 162 that are able to inhibit EZH2 [310].

Determination of the spatial structure of the PRC2 complex allowed scientists to develop a new approach to the creation of small-molecule inhibitors [43, 311, 312].

First, the SAH-EZH2 peptide, which mimics the EED region required for interaction with EZH2, was synthesized [313]. Treatment of cells with SAH-EZH2 impairs PRC2 complex formation, reduces the level of H3K27me3, and inhibits the proliferation of malignant blood and retinoblastoma cells [313, 314].

Second, a region in the EED protein that specifically interacts with the H3K27me3 modification and is important for the recruitment of PRC2 to chromatin was used as a target. Two small-molecule substances have been developed: A-395 and EED226. Their activity against lymphoid tumor cells is comparable to that of EZH2 methyltransferase domain inhibitors [315, 316]. It should be noted that A-395 displays a cytotoxic activity against tumor cells that have acquired resistance to GSK126, which is an inhibitor of the EZH2 methyltransferase domain [315]. Thus, a combination of inhibitors targeting different regions of the PRC2 complex can be used to avoid the emergence of resistance to chemotherapeutic drugs.

Third, the hydrophobic tagging (HyT) method is used to suppress PRC2 activity. In this case, the chimeric molecule is created, one part of which binds to the target protein, and the other one directs the bound complex to proteasome degradation [317]. This method allowed researchers to develop the MS1943 inhibitor, which is specific to EZH2 [318]. MS1943 was shown to suppress the growth of the triple-negative breast cancer MDA-MB-468 cell line, which is resistant to EZH2 methyltransferase domain inhibitors. Thus, inhibition of the methyltransferase activity and degradation of the PRC2 complex can have different therapeutic effects, something that can be implemented in medical practice by using combinations of different drugs.

## CONCLUSION

It has been more than 70 years since the discovery of the *Polycomb *mutation. Tremendous progress has been made in the study of how the PcG/TrxG system functions. The global role of these factors in the transcription regulation and maintenance of cellular homeostasis is becoming clearer. There is also a growing body of data concerning PcG/TrxG dysfunctions in various pathologies. However, a number of questions needing to be addressed for a more complete understanding of the system’s functioning remain open. The details of PcG/TrxG complexe’s recruitment to specific genomic regions remain unclear. The exact contribution of various factors to these processes, such as the activity of specific DNA-binding factors, epigenetic marks, non-coding RNAs, as well as other unknown biological processes, has not yet been established. The increasing complexity of the PcG/TrxG system in the process of evolution from invertebrates to mammals and the emergence of numerous paralogs of these proteins represent a challenge for researchers: to what extent the currently understood composition of protein complexes is characteristic of all types of cells? Are they unique only to a number of tissues and/or developmental stages and do they differ in others? Numerous recent studies assign a crucial role to the spatial organization of genes in the nucleus. These processes were also shown to be closely related to the functioning of PcG/ TrxG complexes. It is important to determine the extent to which the spatial organization determines the functions of DNA regulatory elements or whether it is a consequence of transcriptional complexes’ recruitment to chromatin. Much needs to be done to further elucidate the significance of PcG/TrxG factors in medicine, in the development of improved small-molecule inhibitors, and in the creation of optimal therapeutic protocols. At the same time, despite the tremendous progress achieved in the study of PcG/TrxG proteins in mammals, the *Drosophila *remains an indispensable model organism for studying the details of transcription control by PcG/TrxG proteins.

The emergence of biological methods (genome editing, high-throughput sequencing, mass spectrometry approaches etc.) provides hope that many of these questions will be answered in the future. However, what remains quite clear is that the long road in the study of PcG/TrxG factors is far from being completed.

## Abbreviations

PcG: Polycomb group
TrxG: Trithorax group
PRE: Polycomb Response Element
